# Oncogenic pathways and the electron transport chain: a dangeROS liaison

**DOI:** 10.1038/s41416-019-0651-y

**Published:** 2019-12-10

**Authors:** Vittoria Raimondi, Francesco Ciccarese, Vincenzo Ciminale

**Affiliations:** 1 Veneto Institute of Oncology IOV – IRCCS, Padua, Italy; 2 Department of Surgery, Oncology and Gastroenterology, University of Padua, Padua, Italy

**Keywords:** Cancer metabolism, Oncogenes

## Abstract

Driver mutations in oncogenic pathways, rewiring of cellular metabolism and altered ROS homoeostasis are intimately connected hallmarks of cancer. Electrons derived from different metabolic processes are channelled into the mitochondrial electron transport chain (ETC) to fuel the oxidative phosphorylation process. Electrons leaking from the ETC can prematurely react with oxygen, resulting in the generation of reactive oxygen species (ROS). Several signalling pathways are affected by ROS, which act as second messengers controlling cell proliferation and survival. On the other hand, oncogenic pathways hijack the ETC, enhancing its ROS-producing capacity by increasing electron flow or by impinging on the structure and organisation of the ETC. In this review, we focus on the ETC as a source of ROS and its modulation by oncogenic pathways, which generates a vicious cycle that resets ROS levels to a higher homoeostatic set point, sustaining the cancer cell phenotype.

## Background

Altered reactive oxygen species (ROS) homoeostasis is emerging as an important hallmark of the cancer cell phenotype. These alterations are consistent with the “ROS rheostat” theory,^1^ which states that, depending on their homoeostatic set point, ROS can control different signal transduction pathways, thus acting as either tumour promoters or tumour suppressors. At low/medium levels, ROS enhance mitogenic signalling and cell survival, and may contribute to genetic instability.^2^ It is thus not surprising that most tumours exhibit a higher ROS set point compared with their healthy counterparts. On the other hand, excessive ROS levels result in extensive macromolecular damage and engage cell death pathways.

It should be noted, however, that ROS-producing pathways are not the only determinants of the ROS rheostat, and cancer cells commonly increase the fuelling of antioxidant pathways and may cope with oxidative damage by upregulating repair systems.^3^

Mitochondria are an important source of ROS production through the activity of the electron transport chain (ETC).^4^ Although there are also many other important sources of ROS generation (e.g. NADPH oxidases [NOX]),^5^ ETC-derived ROS are pivotal regulators of cell fate, given the central role of mitochondria in life and death.

Electron transfer through the ETC is a tight and efficient molecular machine. Nevertheless, it is estimated that in normal conditions, about 0.2–2% of the electrons that pass through the ETC complexes leak from the system and reduce O_2_, generating superoxide (O_2_^•–^),^6,7^ which is rapidly converted by superoxide dismutases (SOD) into hydrogen peroxide (H_2_O_2_), the most important ROS acting as a “second messenger” in signal transduction due to its relatively long half-life and diffusion across membranes via the aquaporin channels.^8^ The hydroxyl radical (^•^OH) is a highly damaging ROS with an extremely short half-life that is generated from H_2_O_2_ in the presence of iron or copper through the Fenton reaction. O_2_^•–^ can also interact with nitric oxide (NO), generating the reactive nitrogen species (RNS) peroxynitrite (ONOO^−^), which controls signalling molecules through the nitration of tyrosine residues.^9^ For the role of RNS in cancer we refer the reader to recent excellent reviews.^10,11^

It is important to note that many tumours presenting mutations in ETC components show a strong propensity to produce ROS,^12^ supporting the crucial role of this machinery in the modulation of the cancer cell phenotype.

Several signal transduction pathways that control cell turnover are known to be ROS-sensitive (reviewed in ^13–15^). Through the reversible oxidation of cysteine residues to sulfenic acid (SO^–^)^16^ and disulfide bonds,^17^ ROS modulate the activity of redox-sensitive proteins by controlling several aspects of the cancer phenotype. The oxidation of phosphatase and tensin homolog (PTEN) results in AKT-mediated cell survival and proliferation,^18^ while the oxidation of prolyl hydroxylases leads to hypoxia-inducible factor 1α (HIF-1α) stabilisation,^19^ resulting in a profound metabolic rewiring of cancer cells. Notably, O_2_^•–^ may inhibit apoptosis in cancer cells, accounting for resistance to Fas-mediated cell death.^20^ In this regard, decreasing the levels of O_2_^•–^ restores apoptosis in cancer cells overexpressing the anti-apoptotic protein BCL-2^21^ (see below).

In addition, increased mitochondrial ROS levels drive “mitohormesis”, a condition of mild mitochondrial stress that favours pro-survival pathways through the activation of mammalian target of rapamycin (mTOR) complex 1 (mTORC1) signalling, an alteration that is frequently associated with poor clinical outcome of cancer patients.^22^ Notably, the mitochondria-targeted O_2_^•–^ scavenger mitoTEMPO inhibits tumour cell migration and metastasis in mice,^23^ corroborating a crucial role for ETC-derived O_2_^•–^ in tumorigenesis.

In this review, we focus on the ETC as a source of ROS, its alterations in cancer cells and its modulation by oncogenic pathways, highlighting the connection between ETC-derived ROS and the cancer cell phenotype.

## Sites of ROS production in the mitochondrial ETC

Functional electron transport provides the bioenergetic fuelling necessary to sustain tumour initiation, growth and dissemination. However, defects in the ETC may also  favour tumorigenesis.

**Fig. 1:**
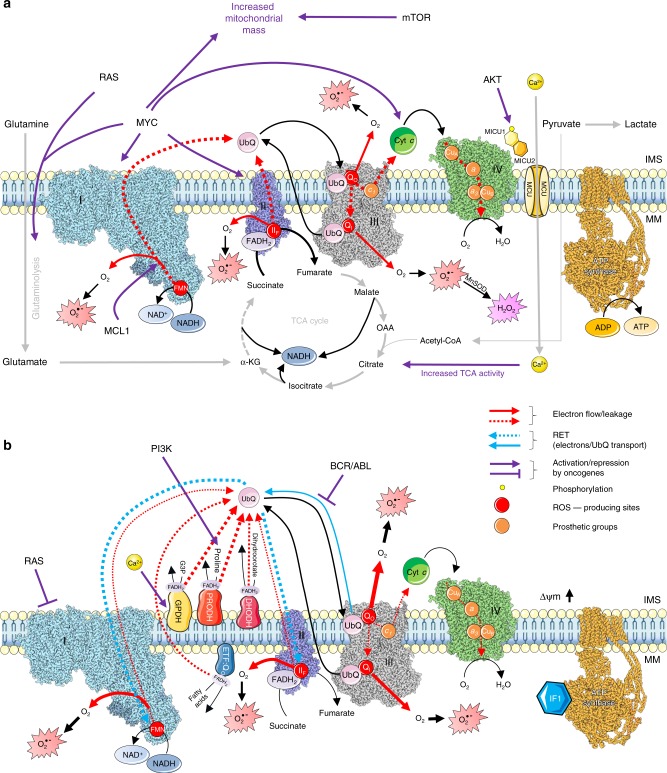
Oncogenes regulate ETC-mediated ROS production. Dashed red arrows indicate electron flow through complexes I (blue), II (purple), III (grey) and IV (green). Solid red arrows indicate electron leakage to O_2_ with consequent generation of O_2_^•–^, which can be converted into H_2_O_2_ by manganese superoxide dismutase (MnSOD). The arrows thickness represents the intensity of reactions/electron flux. **a** Enhanced electron flow through the ETC favours ROS production. RAS and MYC induce glutaminolysis, AKT phosphorylates MICU1 leading to calcium influx, and both these mechanisms promote TCA cycle activity and thus NADH production. MCL1 increases electron flow. mTOR and MYC induce mitochondrial biogenesis (purple arrows). **b** Destabilisation of electron flow across the ETC enhances electron leakage and ROS generation. RAS induces a decrease in complex I activity, thus disrupting the respirasome; PI3K increases proline fuelling through PRODH, bypassing complex II and destabilising electron flow; AKT-driven calcium influx promotes the activity of GPDH, thus inducing RET (light-blue arrows); BCR/ABL impairs electron transfer from complexes I and II to complex III (purple arrow and blunted arrows). IF1 blocks ATP synthase (yellow structure), leading to hyperpolarisation and inducing RET. Both the increase and destabilisation of electron flow drive ROS generation. IMS: intermembrane space. MM: mitochondrial matrix. G3P: glycerol 3-phosphate. Δψm: mitochondrial transmembrane potential. The structures of ETC complexes were obtained from RCSB Protein Data Bank: complex I (5XTD^204^), complex II (3AEF), complex III (5XTE^204^), complex IV (5Z62^205^) and ATP synthase (5ARA^206^). Structures were prepared with UCSF Chimera.^207^

The ETC is responsible for the transfer of electrons from reduced nicotinamide adenine dinucleotide (NADH) and flavin adenine dinucleotide (FADH_2_), produced in the tricarboxylic acid (TCA) cycle, to oxygen, across complexes I, II, III and IV. This electron flow provides energy for proton translocation, thus generating a transmembrane potential (Δψ_m_) that drives ATP synthesis by the ATP synthase complex (Fig. 1). The proton gradient may be either dissipated or increased by the ATP synthase complex when working in its ATP-producing or ATP-consuming mode, respectively. In mammals, complexes I and III have been identified as the most relevant sites of ROS production within the ETC.^24,25^

Complex I (or NADH-ubiquinone oxidoreductase, Fig. 1) oxidises NADH to NAD^+^ and transfers electrons to the carrier ubiquinone (UbQ, also known as coenzyme Q10), resulting in its reduction to ubiquinol (UbQH_2_) and the translocation of protons across the inner mitochondrial membrane (IMM).^26^ During this process, single-electron reduction of O_2_ can occur, leading to the generation of O_2_^•–^, which is converted into H_2_O_2_ by manganese superoxide dismutase (MnSOD). H_2_O_2_ can diffuse across the membrane and be reduced to H_2_O by mitochondrial and cytoplasmic peroxiredoxins, catalases, thioredoxin peroxidases and glutathione peroxidases.^27^ Mammalian complex I contains 44 subunits, seven of which (ND1, ND2, ND3, ND4, ND4L, ND5 and ND6) are coded by the mitochondrial genome, and the others by the nuclear genome.^28^ ROS can be generated when the electrons are transferred from NADH to UbQ through the flavin (I_F_) and the UbQ-reducing (I_Q_) sites in complex I.

Mutations in complex I components may result in increased production of O_2_^•–^, which may sustain ROS-dependent oncogenic pathways and induce damage of mitochondrial DNA.^29^ In addition to affecting supercomplex assembly (see below), defects in ND2 subunit are known to promote tumorigenesis and metastasis in breast, pancreatic and oral cancers, and head and neck carcinomas.^30^ Similar phenotypes were also reported for mutations of the ND6 subunit in lung cancer,^31^ and ND4 in acute myeloid leukaemia^32^ and in glioblastoma.^33^ These mutations are probably associated with increased ROS generation, which in turn selects for increased fuelling of scavenging pathways and enhanced production of NADPH by the folate pathway, resulting in the promotion of metastatic dissemination.^34^ Wheaton et al.^35^ also demonstrated the importance of complex I in cancer progression by using its inhibitor metformin, which reduced tumorigenesis in vitro and in vivo. Metformin acts upstream of the ROS-producing I_F_ site, inhibiting ROS generation from complex I and reducing electron flow to complex III, thus decreasing the production of ROS from it. In hypoxic conditions, NADH depletion and supercomplex disassembly (see below) hamper complex I activity, and ROS generation occurs mainly from complex III.^36^ However, during hypoxia, complex I may produce ROS by reverse electron transfer (RET, see below). In contrast, the inhibition of complex I at the I_Q_ site by rotenone increases ROS generation and induces apoptosis of breast cancer cells through the activation of the c-Jun N-terminal kinase (JNK) and p38 pathways.^37^

Complex III (or ubiquinol–cytochrome*c* oxidoreductase, Fig. 1) transfers the electrons received by UbQH_2_ from complex I and complex II to cytochrome *c* and couples this reaction with proton translocation across the IMM.^38^ Complex III is a dimer containing three highly conserved core subunits: cytochrome *b* (containing two haem groups—*b*_H_ and *b*_L_), cytochrome *c*_1_ and the Rieske iron–sulfur protein (ISP, with an Fe–S cluster). Eight additional subunits in the complex are required for its assembly, stability and regulation.^39^ The reaction catalysed by complex III, known as the “Q-cycle”,^40^ involves two different binding sites on cytochrome *b*, Q_O_ for UbQH_2_ and Q_I_ for UbQ.

In the IIIQ_O_ site, electrons may leak and interact with O_2_, by producing O_2_^•–^ both in the matrix and the intermembrane space (IMS), where it is converted into H_2_O_2_ by SOD.^41,42^ H_2_O_2_ can cross the outer mitochondrial membrane and reach the cytoplasm, where it can act as a signalling molecule. Several studies reported that the inhibition of Q_I_ site with antimycin A blocks Q_O_–Q_I_ electron transfer, resulting in increased electron leak and consequent ROS generation at the IIIQ_O_ site.^43,44^ Although it is reported that the inhibition of binding of UbQH_2_ to the Q_O_ site by stigmatellin and myxothiazol prevents the production of ROS in complex III,^43^ it is plausible that a block in electron transfer across complex III could induce RET (see below) and ROS production.

In the context of cancer progression, it was shown that ubiquinol–cytochrome*c* reductase core protein II (UQCR2), an important subunit of complex III, is upregulated in human tumours, including lung adenocarcinoma and breast cancer.^45^ Interestingly, UQCR2 negatively regulates p53 levels by inducing its degradation through the interaction with PHB, a p53 chaperone. This blunts p53/p21-mediated cell-cycle arrest and senescence, thus promoting tumorigenesis. Although ROS generated from complex III might stabilise p53, the overexpression of UQCR2 can revert this effect, by supporting tumour cell growth and dissemination. The role of UQCR2 in tumorigenesis is supported by a study by Shang et al.,^46^ who demonstrated a positive correlation between UQCR2 overexpression, tumour progression and poor prognosis in colorectal cancer. Based on these findings, the authors proposed UQCR2 as a prognostic biomarker and therapeutic target.

A similar role is attributable to ubiquinol–cytochrome*c* reductase hinge (UQCRH), another subunit of complex III that is overexpressed in lung adenocarcinoma^47^ and in hepatocellular carcinoma.^48^ UQCRH regulates electron transfer from cytochrome *c1* to cytochrome *c*, and its upregulation results in enhanced ROS production.^47^ In hepatocellular carcinoma, UQCRH overexpression is accompanied by the upregulation of two other subunits of complex III, the previously described UQCR2 and UQCRB. Park et al.^48^ observed that high expression of these proteins correlates with poor prognosis, suggesting that they may serve as useful cancer outcome biomarkers.

These observations suggest that inhibitors of ETC complexes could present a new opportunity for cancer therapy. Consistent with this notion, complex III inhibitors, such as myxothiazol and antimycin A, block the reoxidation of UbQH_2_, by impairing the UbQ-dependent processes in breast cancer cells.^49^ It is thus likely that inhibition of complex III might revert electron flow, thus inducing massive ROS generation from complex I and leading to cell death.

Although complex III was previously considered to play a prominent role in ROS production, Liu et al.^50^ proposed that the FMN group in complex I is the leading site in the ETC responsible for generating H_2_O_2_ from succinate (through RET, see below) and malate/glutamate.

Small amounts of ROS are also produced in the flavin-reducing site (II_F_) within complex II (or succinate–ubiquinone oxidoreductase, Fig. 1),^51^ a heterotetramer composed of two hydrophilic subunits (SDHA, SDHB) and two hydrophobic subunits (SDHC, SDHD).^52^ The SDHA catalytic domain contains the prosthetic group FAD, which is reduced to FADH_2_ as a consequence of the oxidation of succinate to fumarate, thus generating electrons that are mobilised to the SDHB subunit. Here, three Fe–S clusters promote electron transfer to the SDHC and SDHD subunits associated with the haem and UbQ prosthetic groups. Haem favours the reduction of UbQ to UbQH_2_, which shuttles from complex II to complex III, where it is re-oxidised before returning to complex II.^53^ Although complex II is not involved in the generation of the transmembrane potential, it has an important role in transferring electrons, bypassing complex I.^54^ Electrons leaked by FADH_2_ in the II_F_ site can contribute to ROS generation, although this event can be decreased when the binding of malate or succinate blocks the site’s access to O_2_.^55^ Many diseases related to mutations in complex II proteins are characterised by increased ROS production from the II_F_ site. An example is represented by the loss of complex II function, which occurs in hereditary paraganglioma–pheochromocytoma, renal cell carcinoma and gastrointestinal stromal tumours. These mutations corrupt complex II’s electron transport activity, leading to succinate accumulation, increased ROS generation and decreased ATP production through oxidative phosphorylation (OXPHOS).^56,57^ The frequent loss of complex II subunit expression in cancer (in particular SDHB) suggests that these subunits might have tumour-suppressor functions; indeed, the accumulation of succinate activates the HIF-1α oncogenic pathway.^58^

On the other hand, complex II is overexpressed in haematopoietic stem and progenitor cells compared with differentiated cells. In this cell system, a high ratio between complex II and ATP synthase maintains an elevated Δψ_m_, whose dissipation compromises the self-renewal potential of stem cells. Moreover, high expression of complex II in haematopoietic stem cells is associated with low ROS generation.^59^ The knowledge that cancer stem cells also show decreased levels of ROS^60^ may suggest that a high complex II/ATP synthase ratio could also preserve the cancer stem-cell reservoir.

The last enzyme of the ETC, complex IV (or cytochrome*c* oxidase, Fig. 1), catalyses the reduction of O_2_ to H_2_O. In mammals, it is a 14-subunit complex containing three mitochondrial-encoded subunits (I, II and III) and 11 nuclear-encoded regulatory subunits. Two haem groups, cytochromes *a* and *a*_3_, and two copper centres, Cu_A_ and Cu_B_,^61^ together with a binding site for cytochrome *c* constitute the electron transport machinery of complex IV. Four electrons from four cytochrome*c* molecules are transferred to the Cu_A_ centre and then to the cytochrome *a* and the *a*_3_-Cu_B_ binuclear centre, by reducing Fe^3+^ and Cu^2+^ to Fe^2+^ and Cu^+^. These reactions are accompanied by the passage of four protons across the IMM and the reduction of O_2_ into two molecules of H_2_O, in a very rapid reaction that limits the generation of reactive intermediates.^62^ Although it is not directly involved in ROS production, complex IV activity affects the overall electron flow, with an impact on the electron leakage by previous complexes, and co-operates with oncogenes, such as BCL-2 (see below), to support tumorigenesis. Mutations in subunits I and II of complex IV have been  reported in epithelial ovarian cancer, prostate cancers and acute myeloid leukaemia. Interestingly, the expression of complex IV is enhanced by p53, and the frequent co-mutation of complex IV and TP53 in acute myeloid leukaemia patients correlates with worse prognosis,^63^ probably due to increased damage of mitochondrial DNA and mitochondrial dysfunction.

## Organisation of the ETC

Several studies of the organisation of the respiratory complexes have revealed the crucial role of the accessory assembly factors in ETC function and in preventing ROS production resulting from disassembled OXPHOS subunits.^64^ The “plasticity model”, proposed by Schägger and Pfeiffer,^65^ describes the coexistence of both independent and associated complexes. In particular, complexes I, III and IV (less frequently, complex II) assemble in supercomplexes.^66,67^ While complex I is mainly found associated in supercomplexes, 70% of complex III and 15% of complex IV units are associated with supercomplexes.^68^ In higher eukaryotes, the most frequent supercomplex, known as the respirasome, is composed of one complex I unit, two complex III units and one complex IV unit (I1III2IV1).^69^ Although the mechanism governing the assembly of the respirasome is not well understood,^66,70^ there is evidence that complexes III and IV are able to form autonomously, while mature complex I exists only in the respirasome^71^ and affects its correct assembly. Several studies showed that mitochondrial disorders that reduce the abundance of complex III or IV are often combined with an impairment in complex I expression.^72–75^ Supercomplex assembly optimises the structural proximity of UbQ with complexes I and III^76^ and the channelling of electrons to UbQ and cytochrome *c*, thus enhancing the efficiency of electron transfer among the complexes and reducing ROS generation.

However, mitochondrial remodelling during apoptosis or in hypoxia-induced acidification of the mitochondrial matrix impairs the assembly of supercomplexes.^77,78^ Supercomplex assembly is also connected to OMA1-dependent remodelling of the mitochondrial cristae.^79^ The plasticity of ETC organisation can also fine-tune the production of ROS, and consequently, the activity of ROS-sensitive pathways,^80^ and influences the adaptation of cancer cells to hypoxia.

The balance between supercomplexes and free complexes can influence cellular metabolism. Although assembly of complex I in supercomplexes promotes electron transport through NADH derived from glucose metabolism, free complex I favours electron transport through FAD-linked pathways^81^ (see below). Indeed, by switching the ETC organisation between free complexes and supercomplexes, cancer cells can tailor their metabolism to different microenvironment conditions.

In several cancer types, loss of supercomplex organisation may favour a metabolic switch towards the Warburg effect phenotype.^82^ Consistent with the central role of complex I in respirasome assembly, cancer cells with mutations in ND2 exhibit a glycolytic metabolic profile; interestingly, these cancer cells also exhibited increased metastatic potential.^30^ In addition, the downregulation of NDUFS1, another complex I subunit, is selected by antiangiogenic therapy in ovarian cancer, leading to a stable glycolytic phenotype and increased aggressiveness.^83^

Although increased ROS levels and metabolic rewiring caused by the loss of supercomplex organisation promote tumorigenesis, some evidence suggests that supercomplex assembly could also be enhanced by oncogenes, thus limiting ROS generation by the ETC. For instance, it has been reported that HER2 can translocate to the inner side of the IMM,^84^ where it could promote increased assembly of supercomplexes. Interestingly, Rohlenova et al.^85^ observed that mitochondrial-targeted tamoxifen (MitoTam), which disrupts supercomplex assembly, impairs electron flow from complex I to complex III, thus increasing ROS generation and cell death in HER2-overexpressing breast cancer cells. It is noteworthy that as the electron flow from FAD-linked enzymes to complex III is not affected by MitoTam treatment, hypoxic adaptation could protect cancer cells from its cytotoxic effect. In addition, KRAS contributes to protect against ROS overload through its involvement in the biosynthetic pathway of cardiolipin (see below), an IMM-specific phospholipid. Cardiolipin is sequestered by supercomplexes, being protected from degradation, and in turn, stabilises supercomplexes, thus decreasing the electron leakage.^86^

## ETC-associated enzymes

Metabolic enzymes that link oxidation of their substrates to the reduction of UbQ represent additional ROS-producing sites connected to the ETC (Fig. 1B).

Proline dehydrogenase (PRODH) is an IMM enzyme responsible for proline catabolism that transfers electrons through FADH_2_ to UbQ.^87^ Proline-derived electrons can leak out to O_2_, thus generating O_2_^•–^. The overexpression of PRODH leads to decreased ETC efficiency and increased ROS generation, due to direct competition for UbQ between PRODH and complex II. This effect may be counteracted by succinate,^87^ due to its higher efficiency in reducing the UbQ pool. Depending on the cell context, PRODH may act as either a tumour suppressor or an oncogenic factor.^88^ In cancer cells, by generating ROS, PRODH may trigger apoptosis and suppress mitogenic pathways (e.g. those triggered by the epidermal growth factor receptor [EGFR] and Wnt–β-catenin).^89^ Moreover, p53 directly upregulates PRODH, which, in turn, induces ROS, DNA damage and promotes  cellular senescence.^90^ Consistent with these findings, many tumour types show downregulation of PRODH expression.^89^ In prostate cancer, CMYC negatively regulates PRODH expression via miR23b*, thus promoting tumorigenesis and tumour progression.^91^ All these effects can be reverted by antioxidants.^88^ However, under specific metabolic conditions, PRODH can take on a pro-survival role; indeed, glucose deprivation and/or hypoxia upregulate PRODH through AMP-activated protein kinase (AMPK), independent of HIF-1α/2α, leading to ROS generation, and consequently, to the activation of autophagy, which, in this context, exerts a pro-survival role. Proline catabolism through PRODH is preferentially used to produce ATP under glucose starvation but not in hypoxia.^92^ These observations indicate that PRODH could modulate ETC activity based on nutrient availability and oxygen tension. Moreover, PRODH overexpression was observed in breast cancer metastases, compared with primary tumour samples, supporting a role for PRODH in metastasis formation.^93^

Mitochondrial glycerol-3-phosphate dehydrogenase (GPDH or GPD2), a component of the glycerophosphate shuttle, is associated with the ETC on the outer side of the IMM. Several functions have been described for the glycerophosphate shuttle, the most important being the metabolism of glycerol 3-phosphate, which connects glycolysis, lipogenesis and OXPHOS. GPDH also has an important role in producing ROS by directly leaking electrons and by inducing RET.^94^ In this regard, a panel of prostate-cancer-derived cell lines was shown to possess upregulated  GPDH activity compared with healthy prostate epithelial cells.^95^ Furthermore, highly proliferating, undifferentiated cancers display higher levels of GPDH activity than more differentiated cancers with low proliferative capacity.^96^

Mitochondrial dihydro-orotate dehydrogenase (DHODH) is a FAD-linked enzyme that mediates the oxidation of dihydro-orotate to orotate, which fuels electrons in the UbQ pool and thus may contribute to O_2_^•–^ production at the Q_O_ site of complex III.^97^ As DHODH is a key component of the pyrimidine biosynthesis pathway, which is required for DNA replication, it has an essential role in cancer cell growth.^98^ Indeed, the oncogenic mitogen-activated protein kinase (MAPK), KRAS and mTOR pathways increase DHODH fuelling by inducing de novo pyrimidine biosynthesis, while MYC increases the expression of DHODH.^99^ Although no mutations in DHODH have been reported in cancer, malignant cells are dependent on its enzymatic activity to sustain pyrimidine synthesis.^100^ In fact, inhibition of DHODH through specific antagonists decreases cell growth in many cancer cell lines, in particular acute myeloid leukaemia, and high expression of DHODH correlates with higher-grade gliomas,^98^ suggesting that DHODH inhibition may be a therapeutic strategy to induce tumour cell differentiation. However, it is important to underline that the catalytic activity of DHODH is strictly connected with a functional ETC, which influences the availability of UbQ. The role of DHODH in ROS production is still controversial, as its inhibition can increase or decrease ROS generation in a context-dependent manner.^98^

Electron transfer flavoprotein:ubiquinone oxidoreductase (ETF:QO) is an IMM protein that contains FAD and an Fe–S cluster. Together with electron transfer flavoprotein (ETF), ETF:QO catalyses the transfer of electrons derived from β-oxidation of fatty acids to the UbQ pool. However, the impairment of electron transfer from FADH_2_ to the Q_O_ site may lead to O_2_^•–^ formation.^101^ ETF:QO activity depends on functional complexes I and III, as they share the acceptor UbQ pool. However, as described above, ETF-mediated electron transfer represents the preferential way to fuel the ETC under chronic hypoxia, as the activity of complexes I and II is impaired. Under low oxygen tension, the activation of HIF-1α induces a metabolic rewiring towards glycolysis and the use of glutamine as a carbon source to synthesise fatty acids,^102^ which are catabolised through β-oxidation to sustain electron flow through the ETF system, thus maintaining ETC function and Δψm. Indeed, the inhibition of fatty acid β-oxidation by etomoxir proved to be an effective strategy to overcome hypoxia-mediated resistance to radiotherapy in cancer cells,^103^ suggesting an important role for ETF:QO in cancer survival under hypoxia. Consistent with this hypothesis, a work by Schuetz et al.^104^ reported that ETF:QO is overexpressed in renal carcinoma and oncocytoma, supporting its putative involvement in tumorigenesis.

## Reverse electron transport (RET)

In addition to the canonical “forward” electron flow through the complexes of the ETC described above, electrons may be transferred from UbQH_2_ back to complex I, with the generation of NADH from NAD^+^. This process, which is termed RET, produces a significant amount of ROS. Although RET is well characterised in vitro, its relevance in vivo has long been controversial, and only recently has experimental evidence started to accumulate in support of its role in cell physiology and pathologic processes.^105^

Several studies indicated that the key conditions that lead to RET are accumulation of reduced UbQ and high Δψm. Electrons deriving from complex II, PRODH,  GPDH, ETF:QO and DHODH may drive over-reduction of UbQ.^106^ Furthermore, all conditions inhibiting electron transport downstream of complex III also increase reduction of the UbQ pool. High mitochondrial transmembrane potential may result from increased H^+^ extrusion by the ETC, by the ATP synthase working in its “reverse” ATP-consuming mode, or through the inhibition of the “forward” depolarising/ATP-synthesising mode of action of the ATP synthase. To this effect, an important role may be played by ATPase inhibitory factor 1 (IF1), which, in its active dimeric form, inhibits both the ATP hydrolase and synthase activities of the complex.^107^ Acidification of the mitochondrial matrix favours inhibition of the ATP hydrolase function by IF1.^108,109^ This mechanism plays an important role in hypoxia by preventing ATP depletion by the “reverse” mode of function of the ATP synthase (reviewed by Campanella et al.^110^).

Most studies investigating the relevance of RET in human pathology have been focused on the tissue damage occurring during ischaemia/reperfusion in heart attack and stroke. Chouchani et al.^106^ recently showed that ischaemic tissues accumulate succinate, which, during reperfusion, is oxidised by complex II, giving rise to RET that produces high levels of ROS, which cause extensive macromolecular damage and trigger cell death. Consistent with this notion, inhibitors of complex I or complex II (e.g. rotenone and dimethyl-malonate, respectively), as well as antioxidants, protect the heart from ischaemia/reperfusion-mediated tissue damage.^106,111^

Although the role of RET in cancer has not been directly investigated, it is now clear that oxygen tension in the tumour microenvironment is subjected to ample temporal and spatial fluctuations as a result of the chaotic organisation of the neoangiogenic process.^112^ The resulting imbalance between oxygen supply and demand suggests that the tumour tissue is constantly subjected to recurrent ischaemia/reperfusion cycles in which RET might play a major role in altering ROS homoeostasis. ROS accumulation in hypoxia/reperfusion could also be attributable to Ca^2+^ overload and alterations in ETC supercomplex organisation.^113^

In addition, several studies have provided strong evidence that IF1 is highly overexpressed in primary samples of human colon, lung, breast and ovarian cancer compared with their normal tissue counterparts,^114^ thus suggesting a potential mechanism controlling RET in cancer cells. Gain-of-function and loss-of-function experiments carried out in different cancer cell lines showed that IF1 enhances glycolytic flux, a finding that is consistent with the inhibition of the ATP synthetic activity of the F_0_F_1_ complex by IF1.^114–116^ Overexpression of IF1 in cancer cell lines also increased the production of O_2_^•–^ in the mitochondrial compartment,^114,115^ an effect that is likely to be related to the mitochondrial hyperpolarisation induced by inhibition of the “forward” activity of the ATP synthase. IF1 overexpression also led to the activation of the nuclear factor κB (NF-κB) pathway resulting in a mitogenic or pro-survival effect, depending on the cell type.^114,115^ The fact that these effects were counteracted by the mitochondrial scavenger MitoQ strongly suggests that they were caused by an increase in ROS in the mitochondrial compartment. The metabolic rewiring and ROS-dependent signalling pathways engaged by IF1 overexpression in cancer cells are consistent with the effects of mitohormesis^107^ and with the finding that mitochondrial ROS produced via RET increase the lifespan of both *Caenorhabditis elegans*^117^ and *Drosophila melanogaster*.^118^ However, the final effect of IF1 on the metabolic profile and redox homoeostasis is likely to be more complex and depends on the relative abundance of IF1 versus ATP synthase as well as the phosphorylation of IF1 on S39 by protein kinase A (PKA), which controls its binding to the ATP synthase.^119,120^ Nevertheless, several studies also suggest that high levels of expression of IF1 are associated with a more aggressive tumour phenotype and poor clinical outcome.^121–123^ In addition to IF1, inhibition of ATP synthase by the TCA cycle substrate α-ketoglutarate may also increase ROS production.^117^

## Regulation of ETC-mediated ROS production by oncogenes

Since it is known that low/medium levels of ROS promote cell proliferation by activating several pathways, such as MAPKs and the phosphoinositide 3-kinase (PI3K)–AKT pathway,^13^ it is not surprising that cancer cells are characterised by sustained ROS production that supports their uncontrolled proliferation.^124^ The activation of oncogenic pathways enhances the production of intracellular ROS, which, in turn, leads to the activation of oncogenes in a vicious circle that boosts cell proliferation and drives aggressiveness of cancer cells, thus affecting the outcome in cancer patients. According to the theory of the ROS rheostat,^1^ the concerted action of oncogenic pathways both on the production and the elimination of ROS plays a central role in rewiring multiple cellular functions that sustain the different phases of tumorigenesis.

Current knowledge suggests two main oncogene-mediated mechanisms that influence ROS production by the ETC: (i) increased fuelling of carbon sources in the TCA cycle, resulting in increased production of NADH and FADH_2_, which augments the number of electrons flowing through the ETC (**Mechanism A**, Fig. 1a); (ii) destabilisation of electron transfer through the ETC, which favours the leakage of electrons at complexes I, II and III (**Mechanism B**, Fig. 1b).

### RAS

The RAS family of small GTPases (KRAS, HRAS and NRAS) transduce external stimuli (e.g. binding of growth factors to their receptors) that promote cell proliferation and survival. Mutations at codon 12, 13 or 61 of *RAS* lead to the constitutive activation of RAS signalling in cancer cells.

The constitutive activation of KRAS in human cancers^125^ orchestrates a profound metabolic rewiring that affects mitochondria, leading to ROS generation through **Mechanism A** (Fig. 1). Oncogenic KRAS signalling promotes the catabolism of glutamine, which fuels the TCA cycle, by enhancing mitochondrial ROS generation, resulting in anchorage-independent growth of colon cancer cells^126^; interestingly, this effect appeared to be mediated by mitochondrial, but not cytosolic, ROS and a functional ETC was required for KRAS-driven lung tumorigenesis in vivo. Anchorage-independent growth was abolished in ρ^0^ cells, in which mitochondrial DNA is absent, while cybrids with mutated cytochrome *b* gene restored the production of O_2_^•–^ and anchorage-independent growth. These observations suggest that the oncogenic potential of KRAS is mediated by O_2_^•–^ production from the Q_O_ site of complex III. Similarly, Liou et al.^127^ observed that oncogenic KRAS induces the transformation of pancreatic acinar cells into pancreatic intraepithelial neoplasia through mitochondrial ROS-mediated activation of NF-κB, which drives transcription of the EGFR and its ligands epidermal growth factor (EGF) and transforming growth factor α (TGFα). Interestingly, the authors also demonstrated that the mitochondria-targeted antioxidant MitoQ prevents the development of pancreatic cancer in mice with KRAS mutations, indicating that the oncogenic activity of KRAS requires the generation of mitochondrial ROS. Similar antitumour effects were observed by Weinberg et al.^126^ by using the mitochondria-targeted O_2_^•–^ scavengers MCP and MCTPO. The importance of ROS in KRAS-mediated tumorigenesis is further corroborated by the fact that the mitochondria-targeted drugs Mito-CP (carboxy proxyl nitroxide) and Mito-Metformin block the proliferation of colon cancer cells.^128^

Son et al.^129^ demonstrated that KRAS-driven pancreatic ductal adenocarcinoma relies on glutamine catabolism to generate aspartate that is fuelled into the aspartate transaminase (GOT1)–malic enzyme 1 (ME1) axis, a major producer of NADPH. In this context, glutamine deprivation results in oxidative stress and decreased tumorigenicity, which is rescued by glutathione and N-acetylcysteine (NAC). It is worth noting that these findings do not contradict observations by Weinberg et al.^126^ and Liou et al.^127^ Indeed, the fact that glutamine is required to maintain endogenous antioxidant systems does not exclude that it also fuels the TCA cycle, thus increasing mitochondrial ROS generation. Consistent with these observations, oncogenic KRAS promotes tumorigenesis through the activation of nuclear factor erythroid 2-related factor 2 (NRF2),^130^ the master regulator of antioxidant responses. In the context of KRAS-driven pancreatic ductal adenocarcinoma with mutant KRAS, glutamine is pivotal both to induce cancer-promoting ROS production and to fuel antioxidant pathways, resulting in an increased homoeostatic ROS set point.

Mutant KRAS (G12V) also translocates to mitochondria and impairs electron transport, thus promoting the production of ROS^131^ through **Mechanism B** (Fig. 1). Baracca et al.^132^ observed that digitonin-permeabilised fibroblasts transformed with KRAS reduced their oxygen consumption rate when supplied with glutamate–malate as the respiratory substrate, suggesting a decrease in complex I activity. Oxygen consumption was not reduced when glutamate–malate was substituted with succinate, suggesting that complex II, III and IV activity was unchanged.^132^ The defect in complex I resulted in inefficient electron transport, due to the loss of supercomplex assembly, an alteration that may be further potentiated by the general effects of ROS on respirasome assembly.^133^

In apparent contrast with these observations, Chun et al.^134^ observed that disruption of oncogenic *KRAS* led to reduced expression of three genes involved in mitochondrial phospholipid synthesis—*ACSL5*, *PCK2*, and *AGPAT7*. The functional consequence of these changes was a decrease in the synthesis of cardiolipin, a phospholipid that favours supercomplex assembly, thus optimising respiration. By promoting the synthesis of cardiolipin in mitochondria, oncogenic KRAS may thus increase the efficiency of electron transport and decrease the production of ROS by the ETC. It is not clear, however, whether all KRAS-mutated tumours exhibit an increase in cardiolipin levels in mitochondria. These apparently paradoxical effects may be explained by the fact that the studies by Weinberg et al. ^126^ and Baracca et al. ^132^ used healthy cells transfected with a construct coding for mutant KRAS, while Chun et al.^134^ used KRAS-mutated colon cancer cells (HCT116) in which mutated KRAS was removed by knockout. In the latter case, the impact of oncogenic KRAS on ETC activity was studied in the context of a heavily mutated genetic landscape (4,288 mutations are reported in COSMIC for HCT116), in which the acquisition of oncogenic KRAS could have a protective role by promoting the synthesis of cardiolipin and reducing the generation of ROS fuelled by other oncogenes. Instead, introducing mutant *KRAS* in normal cells permitted a more direct investigation of the effects of mutant KRAS on the ETC. Further investigations will be needed to establish the role of oncogenic KRAS signalling on supercomplex assembly and respiratory efficiency and its possible impact on cancer.

Recent studies suggest that the effects of KRAS on redox homoeostasis are required for maintaining the cancer phenotype and may thus represent attractive therapeutic targets for KRAS-driven cancers, which still represent a clinical challenge due to the lack of effective therapies. In this regard, Shaw et al.^135^ demonstrated that induction of oxidative stress through the small molecule lanperisone kills mouse embryonic fibroblasts transfected with mutant KRAS and restrains their growth in vivo. Interestingly, Iskandar et al.^136^ observed that hyperactivation of mutant KRAS with the small molecule C1 leads to the activation of the PI3K–AKT pathway, which enhances ROS generation (see below), leading to mitochondrial dysfunction, cell death and blockade of tumours with mutant KRAS. These effects are blunted by NAC, indicating that the generation of ROS through the KRAS–AKT axis is necessary to mediate C1 cytotoxicity, corroborating the feasibility of a ROS-based anticancer strategy to target KRAS-driven tumours.

### MYC

The MYC family of transcription factors (CMYC, LMYC and NMYC) controls cell proliferation and apoptosis by regulating a large number of RNA polymerase I-, II- and III-dependent genes.^137,138^ MYC amplification is commonly observed in neuroblastoma and in breast, ovary, prostate and uterine cancers, while the CMYC-immunoglobulin translocation is a hallmark of Burkitt’s lymphoma.^139^

Like RAS, MYC induces complex metabolic rewiring in cancer cells, which is achieved through the stimulation of glycolysis,^140^ mitochondrial biogenesis^141^ and glutaminolysis.^142^ Li et al.^141^ demonstrated that inducible expression of MYC in the B-cell line P493-6 increases mitochondrial mass and enhances the oxygen consumption rate, an indicator of ETC activity. These effects are in part mediated by the induction of the mitochondrial transcription factor A (TFAM) by MYC,^141^ thus possibly driving ROS production via enhanced electron flow through the ETC.

Observations by Wise et al.^142^ in glioma cell lines indicated that MYC controls a transcriptional programme that promotes the catabolism of glutamine as a carbon source to fuel the TCA cycle, thus sustaining ROS production by the ETC. Vafa et al.^143^ observed that overexpression of CMYC in human fibroblasts induced an increase in ROS levels, which correlated with the formation of foci of DNA damage; the antioxidant NAC reduced both of these effects. As MYC drove cell-cycle entry and proliferation even if DNA was damaged, these observations suggest a mechanism of MYC-induced genomic instability and selection for p53 loss (a frequent alteration in CMYC-driven tumours^144^), both of which fuel clonal evolution and tumour progression. The role of ROS production following MYC amplification is supported by the fact that exogenous antioxidants (vitamin C and Tiron) inhibited transformation of MYC-overexpressing NIH/3T3 fibroblasts.^145^ Moreover, blunting ROS through mitochondria-targeted vitamin E blocked the proliferation and induced cell death in osteogenic sarcoma cells.^146^

In chemotherapy-resistant triple-negative breast cancer, the upregulation of MYC with the anti-apoptotic protein MCL1 selects for a stem-cell phenotype that is dependent on mitochondrial respiration.^147^ Accumulation of MCL1 in the mitochondrial matrix increases the ability of complexes I, II and IV to transfer electrons. The concerted action of MYC on mitochondrial mass and MCL1 on the ETC  resulted in HIF-1α stabilisation, selection of cancer stem cells and resistance to chemotherapy.^147^ However, as in the case for KRAS, the effects of MYC on ROS homoeostasis are a matter of balance, as MYC may also upregulate mitochondrial peroxiredoxin 3 to protect cells from ROS in hypoxia.^148^ Moreover, NADPH production through serine and one-carbon metabolism protects hypoxic breast cancer stem cells from oxidative stress.^149^ This evidence suggests that ROS trigger the selection of cancer stem cells through the upregulation of increased antioxidant defences, which is consistent with the lower ROS set point of cancer stem cells.^60^

MYC may also act through **Mechanism B** by upregulating the expression of several mitochondrial nuclear-encoded proteins, resulting in an imbalance between ETC subunits coded by the nuclear and mitochondrial genomes that leads to the generation of misassembled respiratory complexes.^150^ Interestingly, Herrmann et al.^151^ observed a strong correlation between the progression from normal prostate epithelium to invasive prostate carcinoma with imbalanced nuclear-encoded versus mitochondrion-encoded subunits of complex IV. This suggests that misassembled respiratory complexes promote tumour progression. MYC also affects ROS production by impinging on cancer cell metabolism. In particular, as mentioned above, MYC promotes the expression of a plethora of genes involved in nucleotide synthesis, comprising DHODH,^99^ thus triggering the generation of ROS by destabilising the electron flow across the ETC.

MYC overexpression is associated with an increased proliferation rate in breast cancer,^152^ and MYC amplification in luminal A breast cancer is associated with poor survival and resistance to endocrine therapy.^153^ Although these studies did not evaluate whether ROS are involved in the aggressiveness of MYC-driven breast cancer, it is known that high ROS levels are associated with resistance to endocrine therapy.^154^ The impact of MYC-driven ROS production on the clinical outcome of cancer needs to be further studied in the future. Unfortunately, MYC is still an undruggable target. However, the dependency of MYC-driven tumours on NADPH production through serine metabolism and one-carbon metabolism implies that inhibition of these pathways could be an effective anticancer strategy for patients affected by these malignancies.

### PI3K–AKT–mTOR

Consistent with their role in apoptosis suppression, cell proliferation, metabolism and anabolic reactions, the interconnected PI3K–AKT and mTOR pathways are hyperactivated in almost 40% of all human cancers.^155–157^

As the PI3K–AKT–mTOR pathway plays a pivotal role in the induction of the Warburg effect^158^ and in the inhibition of autophagy,^159^ cancers with hyperactivation of this pathway accumulate dysfunctional ROS-producing mitochondria that are not eliminated by autophagy.

The metabolism of non-essential amino acids is an important feature of metabolic rewiring in cancer cells and in highly proliferating cells. In fact, cancer cells metabolise non-essential amino acids to obtain nucleotides and lipids, preserve redox homoeostasis and control epigenetic regulation.^160^ Among the 11 non-essential amino acids, glutamine, serine and proline play important roles in tumorigenesis. Proline synthesis mediated by Δ^1^-pyrroline-5-carboxylate (P5C) reductases (PYCRs) and proline degradation through PRODH constitute a “proline cycle” between the cytosol and mitochondria.^88^ In *EGFR*-mutated non-small-cell lung cancer, constitutive downstream activation of the PI3K pathway drives proline synthesis, which fuels EGFR-regulated proline oxidation.^161^ The activity of PRODH decreases the efficiency of mitochondrial electron transport, driving the production of ROS from the Q_O_ site of complex III through **Mechanism B**. These findings suggest that proline metabolism could play an important role in non-small-cell lung cancer with *EGFR* mutations.

Although the PI3K–AKT–mTOR pathway is associated with the induction of aerobic glycolysis, Marchi et al.^162^ provided a mechanism through which AKT could mediate ROS generation through **Mechanism A**. These authors observed that mitochondria-localised AKT phosphorylates MICU1, a regulatory subunit of the mitochondrial calcium uniporter (MCU), resulting in the destabilisation of the MICU1–MICU2 heterodimer, thus leading to increased calcium influx in mitochondria.^162^ Decreased expression of MICU1 following its phosphorylation was associated with increased levels of mitochondrial ROS and enhanced in vivo growth of cancer cells. Mitochondrial calcium overload could account for increased ROS generation through mitochondrial dysfunction, which, however, may trigger permeability-transition-pore-mediated cell death.^163^ Alternatively, increased calcium levels in mitochondria could drive ROS production by stimulating enzymes of the TCA cycle and OXPHOS, thus accelerating oxygen consumption and generation of ROS by the ETC^164^ through **Mechanism A**. Moreover, mitochondrial calcium also increases the activity of  GPDH, producing ROS by direct leaking of electrons and by inducing RET towards complex II and complex I^94^ (**Mechanism B**). The activity of the PI3K–AKT–mTOR pathway is controlled by the tumour suppressor PTEN, which is inactivated in several human cancer types. Observations by Marchi et al.^162^ highlight the central role of PTEN status in the AKT-mediated effect on MICU1. Interestingly, ROS inactivate PTEN through oxidation, thus favouring activation of AKT that phosphorylates MICU1 leading to calcium uptake in mitochondria, and calcium drives the production of ROS by the ETC, thus establishing a vicious circle sustaining activation of AKT and boosting tumour progression. In line with this scenario, scavenging mitochondrial O_2_^•–^ through MitoTEMPO blunted the activation of AKT, reverted the Warburg effect and induced death in melanoma cells.^165^ Moreover, Variar et al.^166^ observed that the mitochondria-targeted antioxidant Mito-CP enhances apoptotic cell death in a Burkitt’s lymphoma cell line by decreasing AKT activation and HIF-1α stabilisation under hypoxia, suggesting the possibility to block hypoxic adaptation in cancer cells by decreasing ROS generated by the ETC.

In a study of breast cancer cells, Jin et al.^167^ recently demonstrated that PI3K–AKT-mediated inactivation of glycogen synthase kinase-3β (GSK-3β) through phosphorylation induces an abnormal activity of complexes I and III, thus altering electron flow and enhancing ROS production through **Mechanism B**. The resulting ROS released in the tumour microenvironment impaired the cytotoxic activity of NK cells by oxidising a serine residue in the initiation factor eIF2B, leading to downregulation of NKG2D and its ligands.^167^ These results provide evidence for an AKT/ROS-mediated mechanism to inhibit innate immune response in the tumour microenvironment. Interestingly, inactivation of GSK-3β also leads to stabilisation of CMYC,^168^ which can further enhance generation of ROS by the ETC. It remains to be elucidated whether the inhibition of GSK-3β by AKT requires ROS-mediated PTEN inactivation.

mTOR is a central kinase that integrates energy sensing and anabolic pathways, co-ordinating protein synthesis and cell growth.^169^ Synthesis of novel cellular components is an energy-consuming process; thus, it is not surprising that mTOR promotes mitochondrial metabolism by indirectly increasing the levels of nuclear-encoded mitochondrial proteins. For instance, mTOR promotes the formation of functional complexes between the transcription factor yin-yang 1 (YY1) and its cofactor peroxisome-proliferator-activated receptor coactivator 1α (PGC-1α), which drives expression of many genes encoding mitochondrial proteins, including cytochrome *c*,^170^ resulting in increased mitochondrial respiration and ROS production through **Mechanism A**. Moreover, mTOR co-operates with oestrogen-related receptor α to promote the transcription of genes involved in OXPHOS and in the TCA cycle.^171^ Furthermore, through the inactivation of 4E-BP proteins, mTOR upregulates nuclear-encoded subunits of respiratory complex I and ATP synthase, thus increasing mitochondrial respiration.^172^ Goo et al.^173^ observed that, in the context of PTEN inactivation, hyperactivated AKT is associated with phosphorylation of 4E-BP1, increased activity of complexes I, III and IV and augmented oxygen consumption. These results suggest that mTOR could enhance mitochondrial respiration, and hence, ROS production through **Mechanism A**. Hyperactivation of the PI3K–AKT–mTOR pathway could also result in an imbalance between nuclear-encoded and mitochondrial-encoded subunits of the respiratory complexes, as observed for MYC, leading to the production of misassembled complexes, loss of supercomplexes and increased ROS production through **Mechanism B**.

Given its central role in co-ordinating metabolism, mTOR is at the crossroads between anabolic pathways and mitogenic signalling of cancer cells. mTORC1 phosphorylates S6K1, which activates carbamoyl-phosphate synthetase 2, aspartate transcarbamoylase, dihydro-orotase (CAD) through phosphorylation on S1859. CAD is a rate-limiting enzyme as it catalyses the first three steps of de novo pyrimidine synthesis, by generating dihydro-orotate from glutamine,^174^ thus fuelling DHODH, which produces ROS. Notably, the oxidation of dihydro-orotate to orotate integrates nucleotide synthesis, which is required to sustain the uncontrolled proliferation of cancer cells, with ROS production by the ETC, which drives cancer initiation and progression. Besides mTOR, KRAS, MYC, AKT and other oncogenes also converge on pyrimidine synthesis. Interestingly, Hail et al.^175^ observed that inhibition of DHODH through terifluonomide decreases mitochondrial ROS levels and has a cytostatic effect in prostatecancer cells. Future studies should be aimed at investigating whether a decreased nucleotide pool or decreased ROS levels may account for the anticancer activity of DHODH inhibitors, as well as the role of DHODH-produced ROS in cancer development.

The hyperactivation of the PI3K–AKT–mTOR pathway is a marker of poor prognosis in several human cancers, such as oesophageal squamous cell carcinoma^176^ and breast cancer.^177^ Yu et al.^178^ recently observed that pterostilbene, an antioxidant compound primarily found in blueberries, slows down the progression of mantle cell lymphoma by targeting the PI3K–AKT–mTOR pathway. However, pterostilbene also directly affects apoptosis and the cell cycle, thus rendering the interpretation of these results more complex; the impact of ROS on cancer in the context of PI3K–AKT–mTOR hyperactivation thus deserves further investigation.

### BCR/ABL

The t(9,22) translocation that is pathognomonic of chronic myeloid leukaemia gives rise to the Philadelphia chromosome and to the chimaeric gene *BCR*/*ABL*, coding for a constitutively active tyrosine kinase that transduces mitogenic and anti-apoptotic signals to cancer cells.^179^

The BCR/ABL fusion protein promotes the production of ROS by the ETC partly through the activation of the PI3K–mTOR pathway. Kim et al.^180^ observed that glucose metabolism is involved in the generation of ROS in BCR/ABL-transformed cells. Treatment with 2-deoxyglucose, the BCR/ABL inhibitor imatinib mesylate or rotenone reduced the production of ROS. The same effect was obtained with wortmannin and rapamycin, which inhibit PI3K and mTORC1, respectively, indicating the tight connections among BCR/ABL, PI3K, mTOR, glucose metabolism and ROS production by the ETC through **Mechanism A**. The decrease in ROS levels following rotenone treatment suggests the involvement of RET (**Mechanism B**) in ROS generation by BCR/ABL. Direct action of BCR/ABL on the activity of the ETC has also been observed.^181^ Nieborowska-Skorska et al.^181^ observed a sharp decrease in electron flow rates between complexes I and II and between II and III, with an increase in O_2_^•–^ production through **Mechanism B**, in BCR/ABL-expressing myeloid precursors. This ROS production was decreased by the mitochondria-targeted antioxidant MitoQ and sustained by complex III, as demonstrated by the rescue effect of the complex III inhibitors myxothiazol, stigmatellin and antimycin A. The small GTPase Rac2 was found to promote ROS generation by complex III, as Rac2 knockout substantially reduced mitochondrial O_2_^•–^ levels and oxidative stress in BCR/ABL-expressing cells.^181^ The authors noted that this mechanism underlies ROS production by the ETC in leukaemia cells with a variety of genetic alterations (FLT3–ITD, TEL–ABL1, TEL–JAK2, TEL/PDGFβR, TEL–TRKC, BCR–FGFR1 and mutated *JAK2*). These observations indicate that different types of leukaemia cells may promote genomic instability and progression through the activation of Rac2, which interferes with electron transport from complexes I to III and II to III, ultimately inducing leakage of electrons from complex III and O_2_^•–^ generation.

### BCL-2

The B-cell lymphoma-2 (BCL-2) family includes several proteins that counteract intrinsic apoptosis by binding pro-apoptotic proteins along the IMM.^182^ Many tumour types, including breast cancer, prostate cancer, B-cell lymphomas and colorectal adenocarcinomas, display BCL-2 overexpression.^182,183^ Several lines of evidence suggest that the upregulation of BCL-2, besides directly blocking apoptosis, creates a pro-oxidant state that promotes cell survival. Chen and Pervaiz^184,185^ showed that the overexpression of BCL-2 in leukaemia cells regulates mitochondrial respiration by affecting ETC activity through direct interaction of BCL-2 with the complex IV subunits Va and Vb, by promoting correct assembly of the complex in the IMM and thus upregulating its activity. Based on these findings, the authors suggested that BCL-2 increases ETC activity and O_2_^•–^ production, leading to a pro-oxidant milieu that favours cell survival. Interestingly, during hypoxia, in the presence of BCL-2, the relative abundance of the subunit Va is higher than that of subunit Vb, leading to reduced complex IV activity and mitochondrial respiration, which, in this context, maintains the mitochondrial redox state unchanged, thus preventing oxidative stress that would otherwise favour cell death.^185^ This scenario is supported by the observation that decreasing levels of O_2_^•–^ trigger apoptosis in BCL-2-overexpressing cancer cells^21^ and suggests that the balance between O_2_^•–^ and H_2_O_2_ could modulate cancer cell survival through O_2_^•–^-mediated inhibition of apoptosis.^186^ Furthermore, the establishment of a mild pro-oxidant milieu prevents the dephosphorylation of BCL-2 on S70 residue, thus improving BCL-2 binding with BAX and cell survival.^187^

## Concluding remarks

In the 1920s, Otto Warburg postulated defective mitochondrial respiration as the cause of cancer.^188^ Although the discovery of genetic alterations in oncogenes and tumour-suppressor genes changed our perspective on cancer pathogenesis, many recent studies identified pro-tumorigenic pathways that affect ROS generation by the ETC, thus integrating genetic and bioenergetic alterations of cancer cells in a unified scenario.

The ETC is the principal source of mitochondrial ROS. Activated RAS, MYC, PI3K–AKT–mTOR and BCR/ABL are examples of oncogenic pathways that affect the ETC to enhance ROS production. Oncogenes may control this process through two main mechanisms: increasing TCA cycle fuelling and mitochondrial mass (here defined as **Mechanism A**); increasing electron leakage from the ETC by either altering its organisation (e.g. disrupting the respirasome) or promoting metabolism through ETC-associated enzymes (defined as **Mechanism B**).

It is worth noting that different oncogenic pathways co-operate to produce the cancer phenotype. In fact, KRAS, MYC and PI3K–AKT–mTOR constitute an interconnected network that fine-tunes the generation of ROS by the ETC. mTORC2 hyperphosphorylates AKT, thus enhancing ROS production through the above-mentioned mechanisms and MYC protein levels through the inhibition of GSK-3β. MYC, in turn, can blunt the production of ROS triggered by mTORC1, thus curbing ROS overload in cancer cells. In this regard, Hartleben et al.^189^ observed that MYC-driven upregulation of tuberous sclerosis complex 1 (TSC1) in Burkitt’s lymphoma cells represses the activation of mTORC1, thus limiting the production of ROS by the ETC. In contrast, oncogenic KRAS induces a metabolic shift resulting in enhanced glycolytic flux and lactate production that inhibits the interaction of TSC2 with Rheb, thus leading to mTORC1 activation.^190^ The observation that oncogenic KRAS promotes MYC protein stability^191^ further complicates this scenario. This complex network of interactions may act as a rheostat to optimise a ROS set point that exploits the tumour-promoting activity of ROS while negating their anticancer effects.

One of the most intriguing features of cancer cells is their metabolic plasticity. The recently described hybrid metabolic phenotype, in which the Warburg effect and OXPHOS coexist, provides cancer cells with the ability to adapt their metabolism to different microenvironments.^192^ Mitochondrial ROS play a central role in this process, through the stabilisation of HIF-1α, which promotes glycolysis, and AMPK, which promotes OXPHOS and fatty acid β-oxidation. It is worth noting that the hybrid metabolic phenotype is promoted by MYC and by fatty acid β-oxidation. The importance of β-oxidation suggests a crucial role of the ETC-associated enzyme ETF:QO in tumorigenesis.

Although the mitogenic role of ROS suggests that dietary antioxidants could prove beneficial in cancer prevention,^193^ experimental and clinical evidence indicates that they favour cancer progression^194–196^ and impair the efficacy of chemotherapy and radiotherapy.^197^ Consistent with this notion, non-small-cell lung cancer cells with loss-of-function mutations in the tumour-suppressor liver kinase 1 (LKB1) are more sensitive to oxidative stress.^198,199^ Indeed, LKB1-mutated patients respond better than wild-type patients to therapies with platinum-based anticancer drugs,^200^ which are potent inducers of ROS.^201^ The contrasting effect of untargeted versus mitochondria-targeted antioxidants on tumorigenesis, although not generalisable to all tumour models, further corroborates the concept that tumour progression requires the generation of mitochondrial ROS and the limitation of cytosolic ROS, to maximise mitogenic signalling and avoid oxidative damage. In this regard, the inhibition of endogenous antioxidant systems in order to increase ROS levels above the toxic threshold represents a promising effective strategy to selectively kill cancer cells.^202,203^

The fact that the described oncogenes are deregulated in a high proportion of extremely aggressive cancers, such as non-small-cell lung cancer, pancreatic ductal adenocarcinoma and triple-negative breast cancer, supports a pivotal role for mitochondrial ROS as determinants of the clinical outcome.

Finally, although well characterised in the context of ischaemia/reperfusion models, the role of RET in cancer is still poorly understood and deserves thorough studies. The fact that two common alterations of cancer cells, i.e. transient intra-tumour hypoxia and IF1 overexpression, are potent inducers of RET provides a strong rationale for a crucial role for RET in the rewiring of redox homoeostasis in cancer.

## Data Availability

Not applicable.
